# Exploring novel independent prognostic biomarkers for hepatocellular carcinoma based on TCGA and GEO databases

**DOI:** 10.1097/MD.0000000000031376

**Published:** 2022-10-28

**Authors:** Miaomiao Hou

**Affiliations:** a Microimmune, 3201 Hospital, Hanzhong City, Shaanxi Province, China.

**Keywords:** hepatocellular carcinoma, prognostic biomarkers, weighted gene co-expression network analysis (WGCNA)

## Abstract

Hepatocellular carcinoma (HCC) has become the fifth most common cancer globally, with the second-highest mortality rate and poor survival outcomes. In our research, we aimed to use The Cancer Genome Atlas and gene expression omnibus databases to identify potential genetic biomarkers to predict and improve the survival rate of HCC patients.

**Methods:**

In GSE60502, GSE76427, and GSE84402, we performed differential expression analysis to obtain differentially expressed genes (DEGs). In the The Cancer Genome Atlas database, the FPKM expression profile was subjected to weighted gene co-expression analysis to obtain modules closely related to HCC. We received common genes by intersecting the genes in the module with the differential genes. Then, we fused the common genes’ expression profiles, survival time, and survival status for univariate, Least Absolute Shrinkage and Selection Operator, and multivariate COX regression analysis to obtain prognostic genes. Predictive genes were performed in K–M survival analysis and combined with clinical data for independent predictive analysis.

**Results:**

After differential expression analysis, GSE60502 obtained 1107 DEGs, GSE76427 obtained 424 DEGs, and GSE84402 obtained 1668 DEGs. Through weighted gene co-expression analysis analysis, we can see that the blue and brown modules were closely associated with HCC. After single and multivariate COX regression analysis, we found that suppressor of cytokine signaling 2 (SOCS2) and SERPINF2 were independent prognostic genes for HCC. After survival analysis, HCC patients with high expression of SOCS2 and SERPINF2 had a longer survival time. These 2 genes in normal liver tissues were higher than in HCC at the transcriptional level.

**Conclusion:**

SOCS2 and SERPINF2 were new independent prognostic genes of HCC. So, they may provide new treatment methods and measures for diagnosing HCC.

## 1. Introduction

Primary liver cancer is the fourth leading cause of cancer-related death worldwide, with hepatocellular carcinoma (HCC) accounting for most cases.^[1]^ There are approximately 841,000 new cases and 782,000 deaths each year.^[2]^ Due to the high recurrence rate and metastasis rate, HCC patients are usually diagnosed with a poor prognosis, especially in the advanced stages.^[3]^ Although the great efforts made in the treatment strategies of HCC in the past few years, including surgical resection, liver transplantation, and comprehensive treatment, the 5-year survival rate of HCC patients is still meager.^[4]^ In recent years, high-throughput sequencing and data analysis have gradually become essential tools for biomedical research, identifying biomarkers for prognosis prediction, recurrence monitoring, and clinical stratification.^[5]^ Based on the prediction of patient survival based on predictive biomarkers, clinical practice can better carry out the individualized treatment.^[6]^ Therefore, there is an urgent need to apply to HCC for exploring critical therapeutic targets.

Many studies identify disease biomarkers by screening differentially expressed genes (DEGs).^[7]^ However, many current studies consider the differences in gene expression between different samples, only looking for biomarkers with differential expression as constraints, ignoring the potential association of each gene.^[8]^ The weighted gene co-expression network analysis (WGCNA) no longer only focuses on DEGs but uses information from thousands of genes with the most changes or all genes to identify the set of genes of interest and significant associations with phenotypes.^[9]^ WGCNA can be used to explore the module (cluster) structure in the network, measure the relationship between genes and modules (module member information), explore the relationship between modules (feature gene network), and sort genes or modules (e.g., about their relationship with sample traits). It can identify candidate biomarkers or therapeutic targets.^[10]^ So far, many potential biomarkers have been determined based on WGCNA using sequencing data. For example, Tang et al^[11]^ identified 5 genes as predictive biomarkers for breast cancer. Humans use WGCNA extensively in cancer research.^[12]^ Therefore, we combined differential expression and WGCNA analysis to explore genes associated with prognosis in HCC.

In this study, we employed the TCGA and Gene expression omnibus (GEO) databases to explore the prognostic markers of HCC. As a result, we provided new ideas and methods for the predictive treatment of HCC.

## 2. Materials and methods

### 2.1. Data downloading

We downloaded the FPKM gene expression profile of HCC and the corresponding clinical data from The Cancer Genome Atlas (TCGA) database. There were 424 samples, including 50 normal liver tissue samples and 374 HCC samples. Considering the number of samples, we downloaded the GSE60502, GSE76427, and GSE84402 expression profiles from the GEO database. There were 36 samples in GSE60502, including 18 normal liver tissues and corresponding HCC tissues. There were 167 samples in GSE76427, including 52 normal liver tissues and 115 HCC tissues. There were 28 samples in GSE84402, including 14 normal liver tissues and corresponding HCC tissues. In GSE60502, GSE76427, and GSE84402, their platforms were GPL96 [HG-U133A] Affymetrix Human Genome U133A Array GPL10558 Illumina HumanHT-12 V4.0 expression bead chip and GPL570 [HG-U133_Plus_2] Affymetrix Human Genome U133 Plus 2.0 Array, respectively.

### 2.2. Differential expression analysis

We used the “limma” R package to screen the DEGs between normal liver tissues and HCC tissues in the expression profiles of GSE60502, GSE76427, and GSE84402, respectively. The screening conditions were |logFC|>1 and adj.p.value (adjusted *P* value) < 0.05.

### 2.3. Weighted gene coexpression network analysis (WGCNA)

We constructed weighted coexpression networks on TCGA RNA-seq data using the WGCNA package in R.First; we filtered out outliers in the RNA-seq data. Subsequently, the Pearson correlation matrix constructed a coexpression analysis of paired genes. Next, we built a weighted adjacency matrix using the power function amn = |cmn|b. We chose an appropriate *b* value to enhance matrix similarity and build a coexpression network. We further transformed the adjacency matrix into a topological overlap matrix to detect genes in the network. Finally, average linkage hierarchical clustering was performed based on topological overlap matrix-based dissimilarity, and we constructed a modular dendrogram with over 50 genes for further analysis.

### 2.4. Association analysis of gene modules and clinical features

To determine the importance of each module, we calculated gene significance between sample traits and gene expression. In the principal component analysis of each module, the main components consisted of module eigengenes, whose expression patterns of all genes summarize profile features. We conduct regression relationships between clinical data and gene expression based on *P* values. Furthermore, we defined the correlations between genes in the corresponding modules and gene expression profiles as module membership. Once we selected the module of interest, the gene significance and module membership for each significant gene are determined, and the threshold for cor.

### 2.5. Accessing the common genes

We obtained the common genes with the Venn diagram by taking the intersection of the modular genes and the differential genes obtained from GSE60502, GSE76427, and GSE84402.

### 2.6. Single-factor COX regression analysis

In the TCGA-LIHC FPKM expression profile, we fused the shared genes and the processed clinical information (survival time and survival status) for single-factor COX regression analysis. *P* with less than .05 as a cutoff value to screen genes associated with prognosis.

### 2.7. LASSO regression analysis and multifactor COX regression analysis

Least Absolute Shrinkage and Selection Operator (LASSO) is a strong regularization in many regression analysis methods. Therefore, it can prevent over-fitting in the modeling process of the multivariate COX regression model. we performed LASSO regression analysis on the prognostic-related genes, and the cross-validation method selected the penalty parameter “lambda.” Furthermore, we subjected to multivariate COX regression analysis of the predictive genes based on LASSO regression analysis.

### 2.8. Kaplan–Meier survival analysis

We determined cutoff values based on the median and divided them into low-risk and high-risk groups. Then, we used the Kaplan–Meier survival method with the log-rank test to test for significant differences between the low-risk and the corresponding high-risk groups.

#### 2.8.1. Independent prognostic analysis.

Because the individual characteristics of different patients may affect their survival rate, we combined the prognostic genes obtained by multivariate COX analysis with clinical information (age, gender, M, N, T, and stage) for single and multivariate COX regression analysis.

### 2.9. The UALCAN online tool

We used the UALCAN online tool (http://ualcan.path.uab.edu/) to analyze the expression of independent prognostic genes at the transcriptional level.

## 3. Results

### 3.1. Differential expression analysis

**Figure 1.**
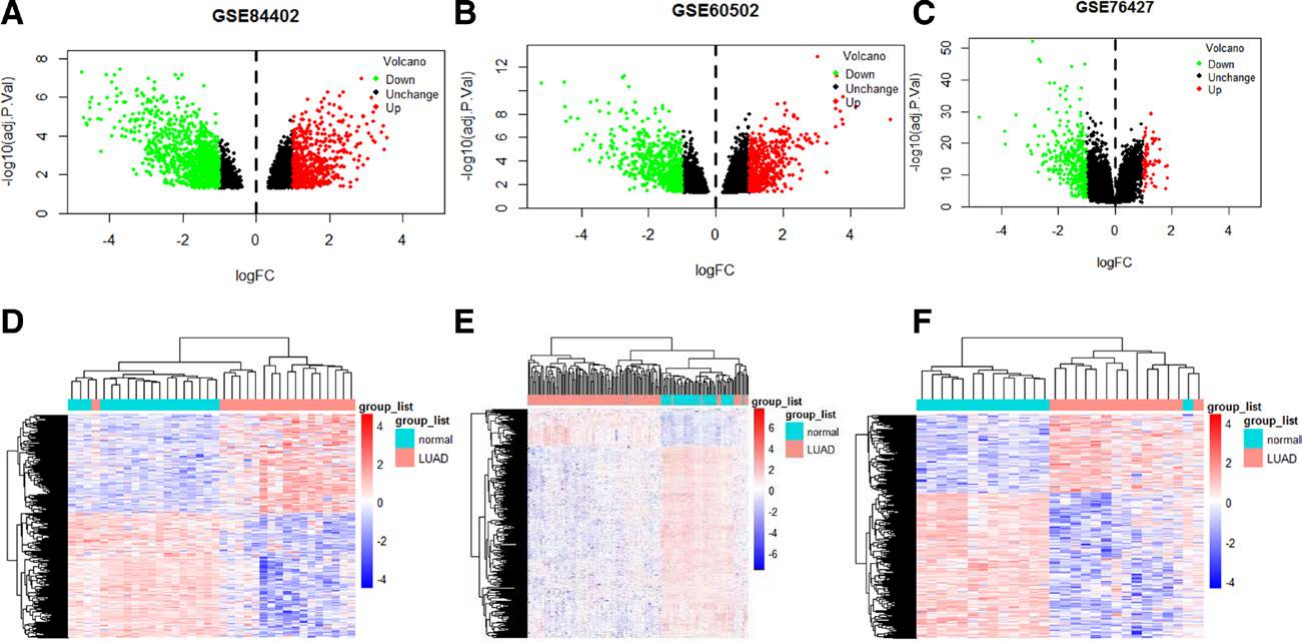
Volcano map and heat map of differentially expressed genes. We drew these graphs by R3.6.1. (A–C) the volcano maps of GSE60502, GSE76427, and GSE84402, respectively. Red represents up-regulated genes, green represents down-regulated genes, and black represents genes that had no significant changes. (D–F) The heat maps of GSE60502, GSE76427, and GSE84402, respectively. Red represents hepatocellular carcinoma tissue, and blue represents normal liver tissue.

After differential expression analysis, GSE60502 obtained 1107 differential genes; GSE76427 obtained 424 differential genes; GSE84402 got 1668 differential genes as shown in Figure 1(A–F).

### 3.2. WGCNA and identification of modular genes

**Figure 2.**
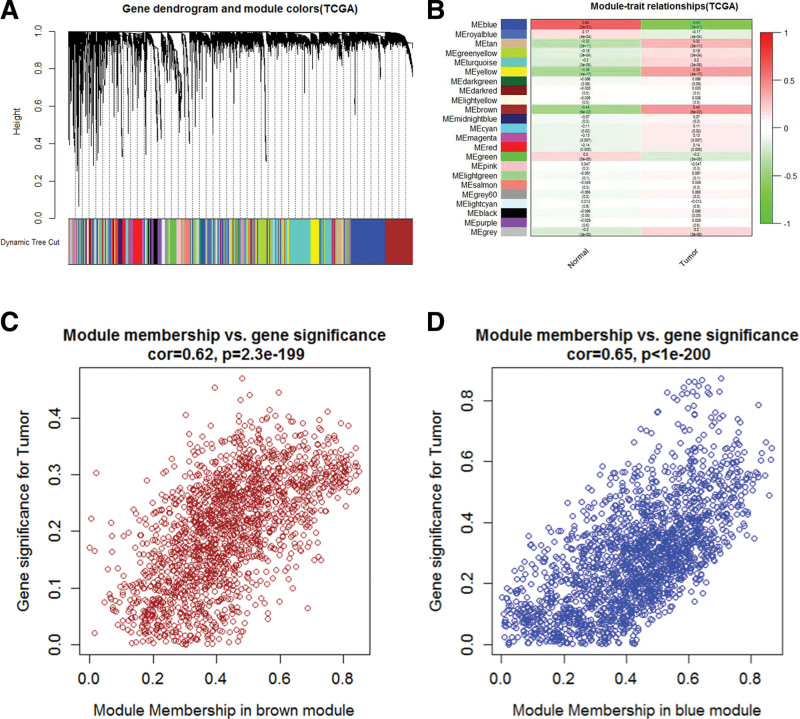
Weighted gene co-expression network analysis. We used the “WGCNA” package in R to draw these figures. (A) Cluster dendrogram of all genes in hepatocellular carcinoma. Each leaf represents a single gene, and each branch represents a coexpressed gene module. (B) Diagram of association analysis between modular genes and clinical traits. (C, D) The scatter plots between the genes of the brown module and the blue module and the occurrence of hepatocellular carcinoma. WGCNA = weighted gene co-expression analysis.

After analyzing the TCGA-LIHC FPKM expression profile through the WGCNA, we obtained 23 gene modules. The blue (cor = −0.65, *P* = 2e−51) and the brown (cor = 0.44, *P* = 6e−22) modules were closely negatively and positively related to HCC, respectively, as shown in Figure 2(A-D).

### 3.3. Accessing common genes

**Figure 3.**
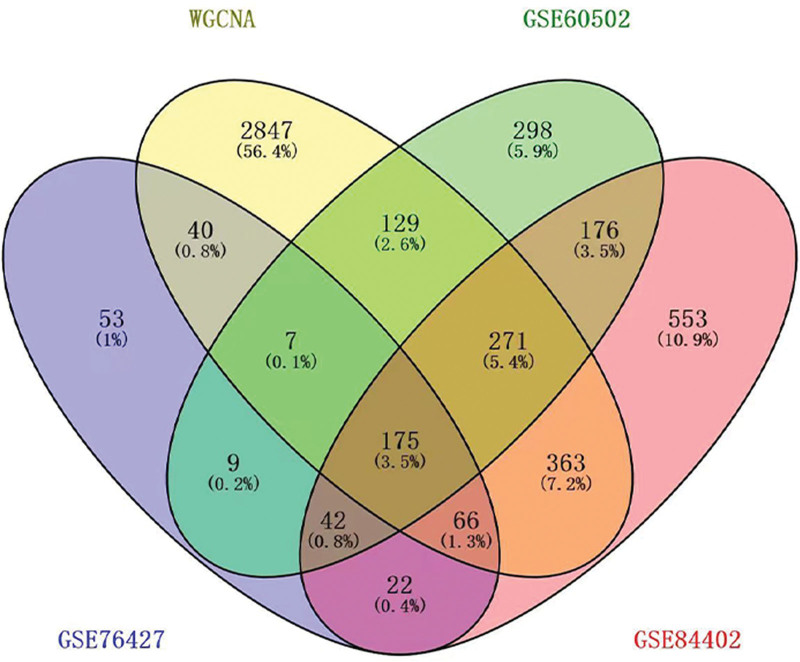
Venn diagram analysis. We used the Venn online website (http://bioinfogp.cnb.csic.es/tools/venny/index.html) to draw Venn diagrams. We intersected the different genes of GSE60502, GSE76427, GSE84402, and WGCNA gene modules (blue and brown modules) to obtain 175 shared genes. WGCNA = weighted gene co-expression analysis.

Using the Venn diagram, we crossed the DEGs of GSE60502, GSE76427, GSE84402, the WGCNA gene modules (blue and brown modules), and finally, we obtained 175 genes as shown in Figure 3.

### 3.4. Single-factor COX regression analysis

**Figure 4.**
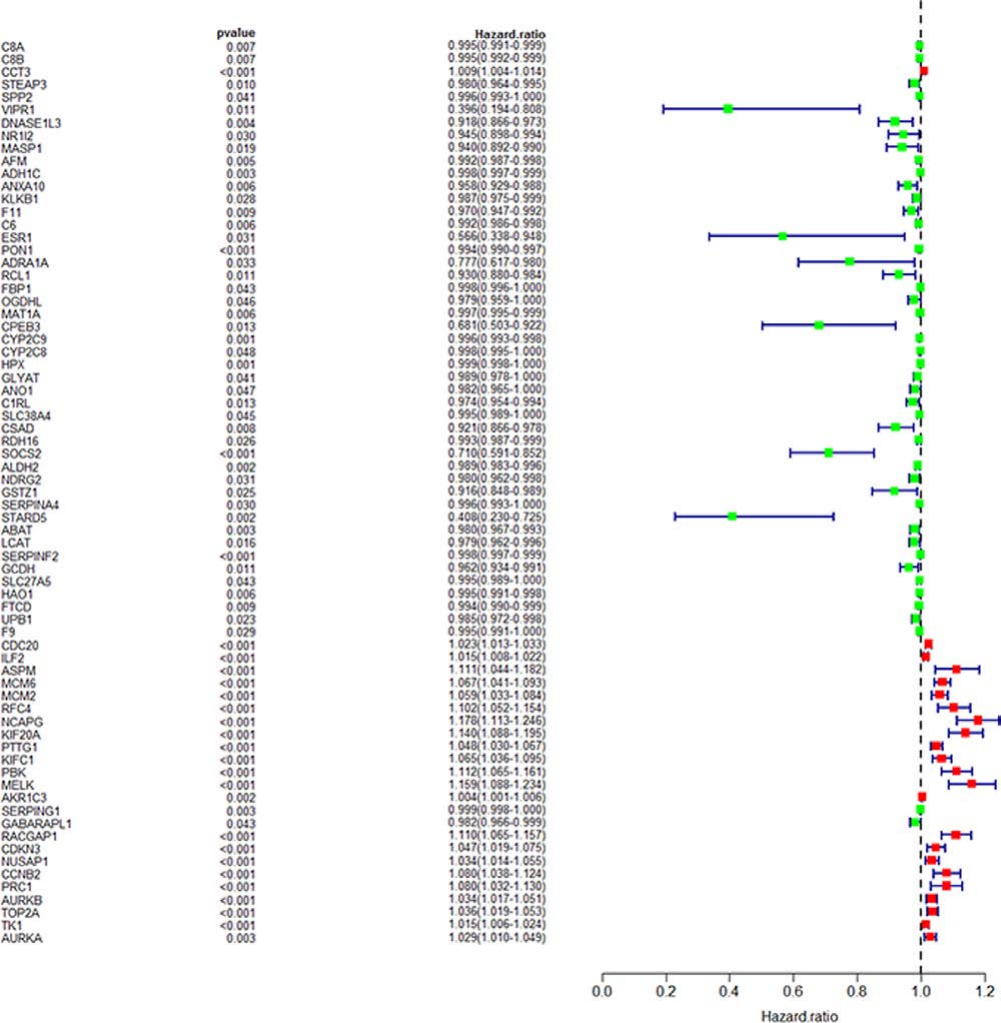
Single-factor COX regression analysis, *P* < .05 indicates genes related to prognosis, HR > 1 indicates genes with poor prognosis, and HR < 1 shows good forecast. We drew the graph by R3.6.1.

In the TCGA-LIHC FPKM expression profile, 175 gene expression profiles were extracted and fused with survival status and survival time for single factor COX regression analysis. As a result, We can obtain 71 prognostic-related genes. Among them, HR > 1 indicates genes with poor prognosis, indicated in red, and HR < 1 indicates genes with good prognosis, indicated in green as shown in Figure 4.

### 3.5. LASSO regression analysis

**Figure 5.**
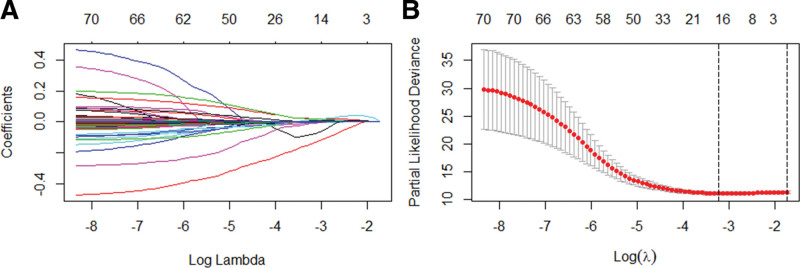
LASSO regression analysis. (A) The LASSO coefficient map of 16 prognostic genes. (B) The cross-validation map. We drew these figures by R3.6.1. LASSO = Least Absolute Shrinkage and Selection Operator.

The 71 prognostic genes were subjected to LASSO regression analysis to prevent the model from overfitting, and we obtained 16 predictive genes as shown in Figure 5.

### 3.6. Multivariate COX regression analysis

**Figure 6.**
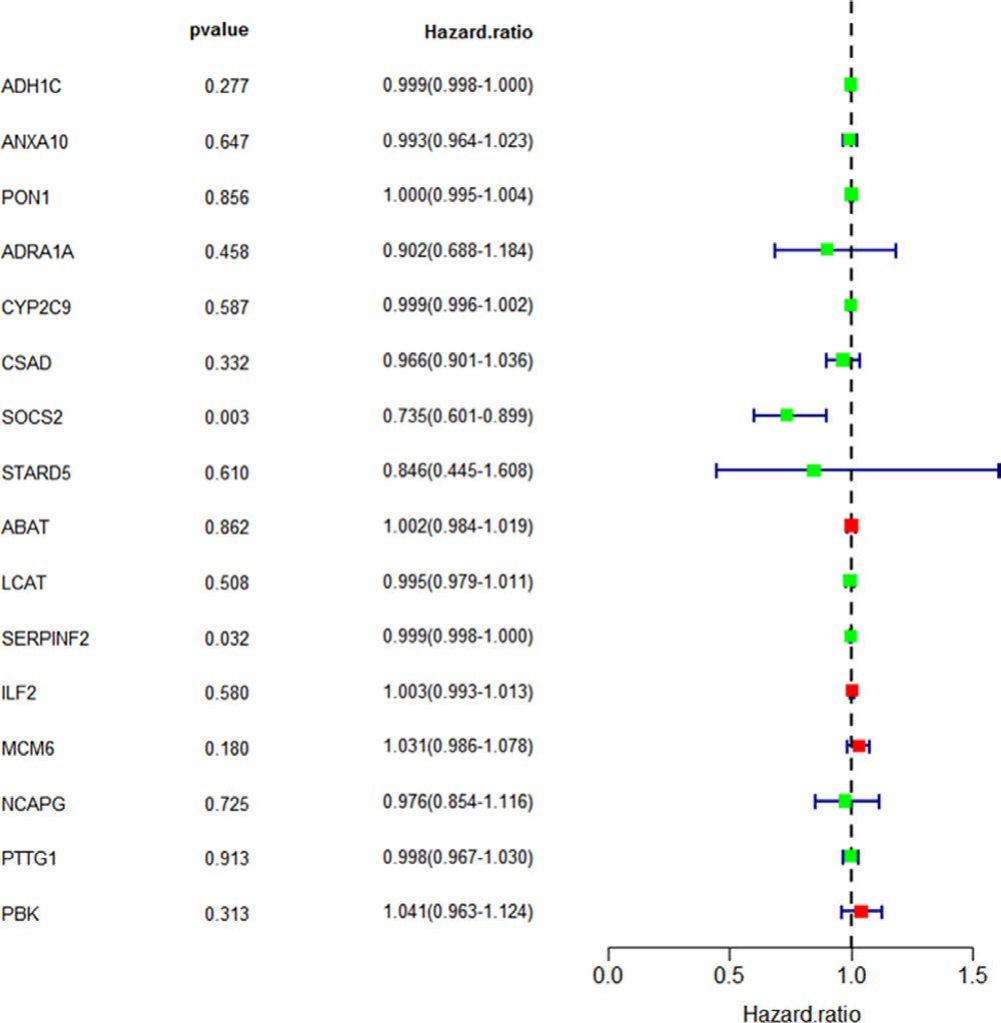
Multivariate COX regression analysis. We obtained 2 good prognostic genes (SOCS2 and SERPINF2). *P* < .05 means significant clinical significance. We drew the graph by R3.6.1. SOCS2 = suppressor of cytokine signaling 2.

After multivariate COX regression analysis, we obtained 2 prognostic genes (SOCS2 and SERPINF2) (Table 1). HR < 1 indicates that they are good prognostic factors for HCC as shown in Figure 6.

**Table 1:** Multivariate COX regression analysis.

Gene	Coef	HR	HR.95L	HR.95H	*P* value
ADH1C	−0.000552213274346255	0.999447939167343	0.998452723299838	1.00044414702435	.277311194940304
ANXA10	−0.00688607488355306	0.993137579802974	0.964312605641592	1.02282418237255	.646789698851114
PON1	−0.000406307713519412	0.999593774818281	0.995211740688495	1.00399510355878	.856165043241428
ADRA1A	−0.102650199890287	0.902442592794433	0.688037755925963	1.1836598010432	.458277742293111
CYP2C9	−0.000866351733832863	0.999134023440478	0.996015406785313	1.00226240477376	.587021671907726
CSAD	−0.0344550510874743	0.966131765282724	0.901163472416593	1.03578386880822	.332007015451825
SOCS2	−0.307724368156762	0.735117911992189	0.601012797114461	0.899146153170578	.00274929018573467
STARD5	−0.167108347718385	0.846107932506687	0.445235893777046	1.6079086242971	.609959183258537
ABAT	0.00154702450410874	1.00154822176383	0.984278878225962	1.01912055892762	.861606813259466
LCAT	−0.00545950362730014	0.994555372378447	0.978590400733917	1.01078080061382	.508464583350205
SERPINF2	−0.0012056036141216	0.99879512283395	0.997696469007586	0.999894986487419	.0317943988923187
ILF2	0.00293866225917739	1.00294298435981	0.992555832429771	1.01343883841187	.580094486966252
MCM6	0.0306295282917842	1.03110343846648	0.985944944030256	1.07833029344565	.180086986839108
NCAPG	−0.0240116341071121	0.976274351611461	0.854035657347507	1.11600915185975	.724980374020265
PTTG1	−0.0017458051815699	0.998255717849862	0.967383085496879	1.03011360562305	.913265827603853
PBK	0.0398326523806144	1.04063661156035	0.963084793700499	1.12443324243429	.31342644187488

SOCS2 = suppressor of cytokine signaling 2.

### 3.7. Survival analysis

**Figure 7.**
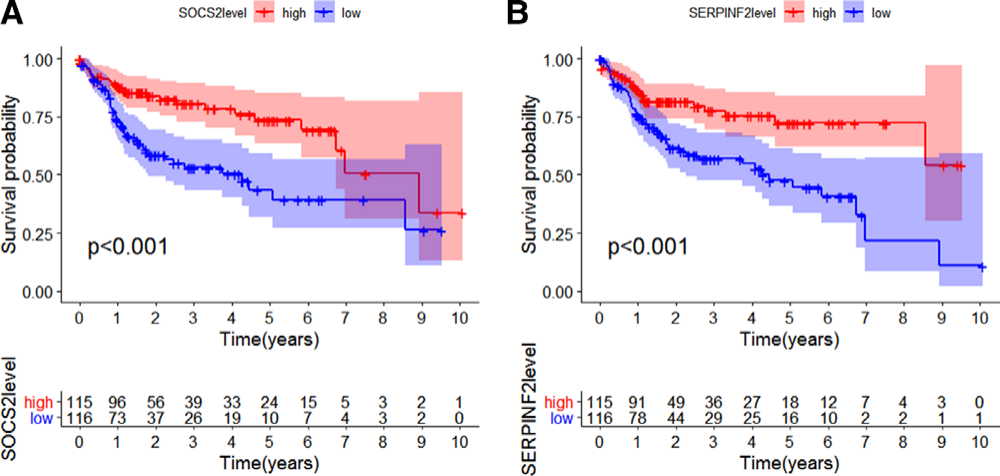
Survival analysis. (A) Survival analysis of SOCS2. (B) Survival analysis of SERPINF2. Red indicates highly expressed genes, and blue indicates low expressed genes. We drew these figures by R3.6.1. SOCS2 = suppressor of cytokine signaling 2.

After survival analysis, patients with high SOCS2 and SERPINF2 expression had a longer survival time than low SOCS2 and SERPINF2 expression HCC as shown in Figure 7(A, B).

### 3.8. Independent predictive analysis

**Figure 8.**
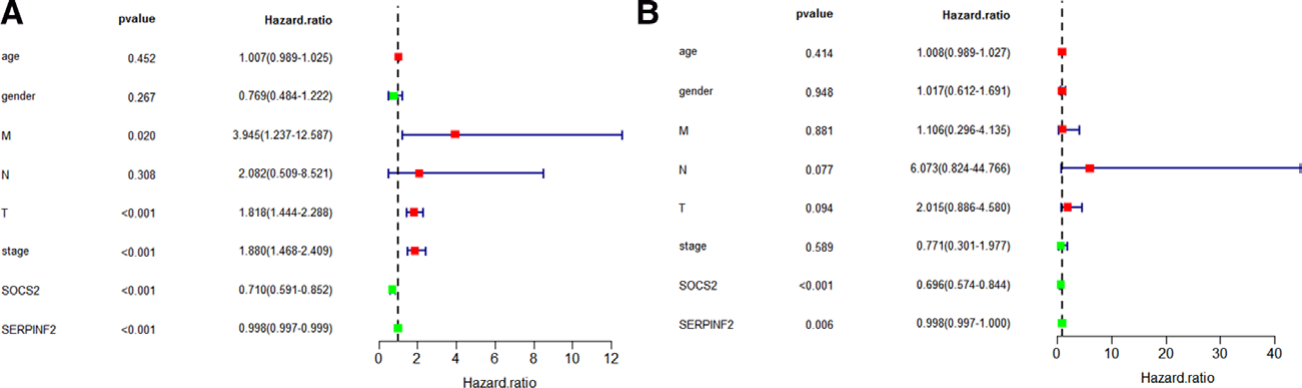
Independent prognostic analysis. (A) Single-factor COX regression analysis. (B) Multifactor COX regression analysis. We drew these figures by R3.6.1.

Combining SOCS2 and SERPINF2 with clinical information (age, gender, T, M, N, stage) for single (Table 2) and multivariate (Table 3) COX regression analysis, *P* < .05 and HR < 1, indicates that these 2 genes were independent good prognostic factors for HCC as shown in Figure 8(A, B).

**Table 2:** Univariate COX regression analysis.

ID	HR	HR.95L	HR.95H	*P* value
Age	1.00691073448016	0.988992697137345	1.02515340117882	.452191064552279
Gender	0.76888170394533	0.483587223724902	1.22248695924642	.266626478127627
M	3.94537172076132	1.23671080782674	12.5865787833916	.020400114473124
N	2.08223370005389	0.508805829906386	8.52131977819091	.307659221420522
T	1.81798318844451	1.44444016379298	2.28812723179065	3.51596564975333e−07
stage	1.88013831278614	1.46750252380055	2.40879999718948	5.91610907391727e−07
SOCS2	0.709521192540666	0.59082912312453	0.852057393518537	.000238703622088336
SERPINF2	0.998162778006546	0.997152188341008	0.999174391880309	.000373577337193434

SOCS2 = suppressor of cytokine signaling 2.

**Table 3:** Multivariate COX regression analysis.

ID	HR	HR.95L	HR.95H	*P* value
Age	1.00778586508156	0.98920527749452	1.02671545832287	.414012436768779
Gender	1.01718943880967	0.611962511639189	1.69074793757296	.947584692717701
M	1.10634173951743	0.296032474166988	4.13465464571977	.880573147800652
N	6.07333155268891	0.823961678557327	44.765863642432	.076731302612951
T	2.01499264628129	0.886447489644571	4.58029991849338	.0944714506121834
Stage	0.771442277011674	0.301021235337217	1.97701396745075	.588893673320392
SOCS2	0.69599493150116	0.574197488890698	0.843627765790377	.000222088666866579
SERPINF2	0.998413604325149	0.997274211944008	0.99955429846962	.00642684230387781

SOCS2 = suppressor of cytokine signaling 2.

### 3.9. The expression of SOCS2 and SERPINF2 at the transcriptional level

**Figure 9.**
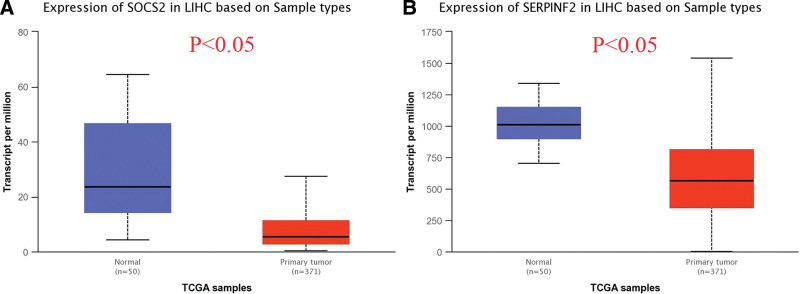
Gene expression analysis at the transcription level. We used the UALCAN (http://ualcan.path.uab.edu/index.html) online tool to show the expression of these 2 genes at the transcriptional level. (A) SOCS2; (B) SERPINF2. Blue indicates normal liver tissues, and red suggests hepatocellular carcinoma tissues. SOCS2 = suppressor of cytokine signaling 2.

Using the UALCAN online tool to analyze the word SOCS2 and SERPINF2 at the transcriptional level, we found that the expression level of these 2 genes in normal liver tissues was higher than that in HCC as shown in Figure 9(A, B).

## 4. Discussion

This study used the TCGA-LIHC FPKM expression profile to perform a WGCNA to obtain the modular genes related to HCC. And we intersected them with the differential genes of GSE60502, GSE76427, and GSE84402 to get 175 shared genes. To obtain genes associated with prognosis in HCC, we linked the expression profiles of 175 genes with survival time and survival status to perform single- and multifactor COX regression analysis. The 2 genes SOCS2 and SERPINF2 were related to the prognosis of HCC. We combined clinical information (stage, M, N, T, gender, and age) to perform single and multivariate COX regression analysis, which showed that these 2 genes were independent prognostic genes for HCC. Survival analysis showed that HCC patients with high expression of SOCS2 and SERPINF2 had a longer survival time. In addition, the transcriptional expression levels indicated that the expression levels of the 2 genes SOCS2 and SERPINF2 in normal liver tissues were higher than in HCC tissues. In conclusion, after a series of bioinformatics analyses, we identified these 2 genes as possible prognostic biomarkers for HCC.

Inhibitors of the cytokine signaling (SOCS) family, such as SOCS1, SOCS2, and SOCS3, be critical negative regulators of the JAK/STAT signaling pathway in HCC. The available evidence suggests that SOCS2 regulates the importance of biological processes in various diseases and cancers by processing the JAK/STAT pathway or other signal transduction.^[13]^ The suppressor of Cytokine Signaling 2 (SOCS2) can inhibit tumor metastasis. the expression of SOCS2 has also been significantly reduced in HCC and is associated with aggressive tumor progression and poor prognosis in HCC patients.^[14]^ Low SOCS2 gene expression is associated with breast, lung, hepatocellular, and ovarian cancer in cancer.^[15]^ Many studies have shown that SOCS2 plays a vital role in inhibiting liver cancer progression.^[16]^ Some people believe that SOCS2 is an independent predictor of survival for breast cancer, colorectal cancer, and HCC patients and that patients with low SOCS2 expression have a shorter survival time.^[17]^ The study found that in various tumor models, the inhibition of SOCS2 induces tumor immune escape by inhibiting the development of Th2 cells and limiting the adaptive antitumor immunity of T cells. patients with SOCS2 overexpression have a better prognosis. these were consistent with our research.^[18]^ SERPINF2, also known as α2-antiplasmin, is a gene encoding plasmin inhibitors that can degrade plasma fibrin and other proteins.^[19,20]^ Plasma SerpinF2 is mainly produced in the liver and kidneys and is reduced in patients with nephrotic syndrome.^[21]^ Chen et al showed that compared with nontumor liver, FGG, FGL1, SERPINF2, and MT1G were downregulation in HCC. It is consistent with current research. Decreased expression of SERPINF2 may lead to an increase in the level of activated plasmin, which will damage the stability of the fibrin bundle and thereby damage the integrity of the extracellular matrix of the liver.^[22]^ Interestingly, the gene interactions between Proc and Serpinc1 and Plg and Serpinf2 are related to liver function and regeneration.^[23]^ However, humans made few studies on the role of SERPINF2 in HCC, so we will further investigate it.

However, our study also has limitations. Mainly based on retrospective analysis of different databases, the expression and function of all identified genes need to be further explored in experiments.

## 5. Conclusion

In this study, we discovered 2 genes (SOCS2 and SERPINF2) related to the prognosis of HCC. So we may provide a clue for the prognosis and treatment of HCC.

## Author contributions

I completed the article by myself (including conceptualization, data curation, formal analysis, methodology, funding acquisition, project administration, supervision, validation, visualization, writing—original draft, writing—review and editing).

Conceptualization, Data curation, Formal analysis, Funding acquisition, Investigation, Methodology, Project administration, Resources, Software, Supervision, Validation, Visualization, Writing – original draft, Writing – review & editing: Miaomiao Hou.
